# Inflammation and Growth in Young Children with Obstructive Sleep Apnea Syndrome before and after Adenotonsillectomy

**DOI:** 10.1155/2014/146893

**Published:** 2014-08-24

**Authors:** Yuval Nachalon, Neta Lowenthal, Sari Greenberg-Dotan, Aviv D. Goldbart

**Affiliations:** ^1^Department of Pediatrics, Soroka University Medical Center, Faculty of Health Sciences, Ben-Gurion University, 84101 Beer Sheva, Israel; ^2^Department of Epidemiology, Soroka University Medical Center, Faculty of Health Sciences, Ben-Gurion University, 84101 Beer Sheva, Israel; ^3^Sleep Wake Disorders Unit, Soroka University Medical Center, Faculty of Health Sciences, Ben-Gurion University, 84101 Beer Sheva, Israel

## Abstract

*Background*. Obstructive sleep apnea (OSA) is associated with growth impairment that usually improves following effective treatment. In this study we investigated the mechanisms underlying the growth processes in young children diagnosed with OSA, before and after adenotonsillectomy (T&A). *Methods*. Young children (6–36 months old) were enrolled and evaluated before and several months after T&A surgery for height, weight, circulating high sensitive C-reactive protein (CRP), and insulin-like growth factor 1 (IGF-1) levels. Caloric intake was assessed by a validated Short Food Frequency Questionnaire (SFFQ). *Results*. Following T&A, children added 4.81 cm and 1.88 kg to their height and weight, respectively (*P* < 0.001 for both) and had a significant increase in BMI Z score (*P* = 0.002). Increased caloric intake of 377 kcal/day was noted (*P* < 0.001), with increased protein and decreased fat intake. The decrease in CRP levels correlated with the increase in body weight in boys (*P* < 0.05, adjusted for caloric intake). *Conclusions*. Adenotonsillectomy results in enhanced somatic growth in young children that correlates with a decrease in systemic inflammation and caloric intake increment. Our findings imply that systemic inflammation may have an important role in this OSA-related morbidity.

## 1. Background

Obstructive sleep apnea syndrome (OSA) is a common problem among children and especially boys, with estimated prevalence of 2-3% [1], with most children being diagnosed at the age of 2–6 years. At night OSA is characterized by interrupted sleep, snoring, and disrupted breathing. At daytime, children present hyperactive behavior, cognitive deficits, and attention problems. Adenotonsillar hypertrophy is considered the leading cause of pediatric OSA and tonsillectomy and adenoidectomy (T&A) is still considered and clinically used as a first line treatment for children with OSA [2].

There is growing evidence on OSA's associated neurobehavioral [3], cognitive [4], cardiovascular [5], and metabolic morbidities [6]. Furthermore, new studies found an improvement in cognitive [7] and cardiovascular [8] functions after T&A.

In the last 30 years there were several studies, as well as case studies, suggesting OSA as a major risk factor for growth failure [9, 10]. These studies were conducted mostly among kindergarten and school children and showed an improvement of somatic growth accompanied with an increased level of IGF-1 and the ratio growth hormone (GH)/IGF-1 after T&A [11, 12]. The very few studies that followed somatic growth in toddlers have also shown an improvement of somatic growth, but none of them have ever tried to estimate the role of growth hormone in the process [11, 12]. There are several possible mechanisms for growth retardation among children with OSA, including increased energy expenditure during sleep due to labored breathing, decreased appetite, and enlarged tonsils that serve as a mechanical barrier limiting the amount of food swallowed. Another possible explanation is interruption in the axis of growth hormone (GH) secretion. GH is secreted from the pituitary gland mainly during slow-wave sleep in a pulsatile fashion. GH secretion positively correlates with IGF-1 and IGFBP-3 levels which triggers bone growth. Notwithstanding the direct effects of GH on growth plate chondrocytes, it is now accepted that the indirect local and systemic effects of GH function in a highly coordinated manner regulate growth plate activities and linear bone growth [13].

In children with OSA there is a decrease in the slow-wave sleep component, and an increase in its percentage of the total sleep time is noted following T&A [14]. This phenomenon was revealed by others and serves as a reasonable explanation to growth retardation before T&A and the improvement in growth after surgery [15].

Another possibility that may explain growth impairment is systemic inflammation.

Linear bone growth is adversely affected in children with chronic kidney disease (CKD) and other chronic inflammatory disorders. CKD patients have been reported to have elevated circulating levels of IL-6 and TNF*α*, similar to patients with OSA [16, 17]. The (GH)/(IGF-1) pathway is anabolic to the skeleton and inflammatory cytokines compromise bone growth through several mechanisms, which include interference with the systemic as well as the tissue-level GH/IGF-1 axis [18]. Recent studies have shown that, in children, OSA is accompanied by systemic inflammation, reflected by increased levels of markers like circulating C-reactive protein (CRP), that decrease after T&A [19]. Interestingly, the role of systemic inflammation, in regard to growth, in such children was not studied so far. Therefore, we assessed growth in OSA young children before and after T&A and evaluated potential mechanisms that may influence growth in these children.

## 2. Methods

The study was approved by Soroka University Medical Center Human IRB Committee, and informed consent was obtained from the legal caretaker of each participant.

### 2.1. Patients

Young children with PSG proven OSA were enrolled and followed prospectively before T&A surgery and 4–6 months afterwards.

Inclusion criteria were children older than 6 and younger than 36 months of age who were diagnosed with an obstructive apnea-hypopnea index (AHI) >5 events/hour of sleep in an overnight polysomnographic evaluation and informed consent from the legal caregiver who agreed was obtained. Exclusion criteria were craniofacial, neuromuscular, syndromatic, or defined genetic abnormalities, any known previous allergies, no upper respiratory infection use of any corticosteroids or antibiotics within 4 weeks preceding the initial sleep study, and any children that had had T&A or adenoidectomy in the past. 

*Overnight Polysomnography.* All participating children underwent polysomnography in our sleep center. No sleep deprivation or sedation was implemented. Children were studied in a dedicated quiet, darkened room with an ambient temperature of 24°C in the company of one of their parents.

Polysomnography was performed with a computerized, commercially available, sleep monitoring system (SensorMedics Inc., Yorba Linda, CA, USA). Data was streamed to an optical disk for later analysis. Polysomnography was performed as previously described [8]. All of the studies were initially scored by a certified technician. The scores were then blindly reviewed by 2 physicians experienced in pediatric polysomnography. Analysis of the polysomnograms was performed with standard techniques [20]. In brief, sleep staging was performed with the standard criteria published by the AASM in 2007 [21] and not by the latest revision of 2012 since we recruited our last child in February 2012. The AHI was defined as the airflow with continued chest wall and abdominal movement over at least two breaths [21]. Hypopneas were defined as a ≥50% decrease in nasal flow with a corresponding ≥3% decrease in SpO_2_ and/or arousal or awakening [21]. We determined the mean and nadir SpO_2_ values. Arousals were defined as recommended by the revised AASM rules [21].

### 2.2. Evaluation of Somatic Growth

In each visit the child was evaluated for height and weight, using the same equipment on both encounters. Anthropometric data was analyzed by the endocrinologist (N.L.) for *Z* score using the software “Growth Analyzer 3.5” of The Dutch Growth Foundation (http://growth-analyser.sharewarejunction.com). *Z* score represents standard deviations gap from the mean to child's age.

### 2.3. Dietary Assessment

A Short Food Frequency Questionnaire (SFFQ) for energy assessment was completed for each child on both encounters. This SFFQ is based on a list of items reported by 160 mothers of infants at the age of 6 to 24 months describing the previous day's food items offered or consumed. This SFFQ was validated in previous studies [22, 23]. All SFFQs were assessed for caloric intake and for the differential percent of protein, fat, and carbohydrates in diet.

It was filled by the primary caregiver at the two encounters.

### 2.4. Inflammatory and Endocrinologic Markers

Circulating hsCRP was measured by means of particle-enhanced immunonephelometry using the BN ProSpec system (Newark, DE). All samples were assessed in duplicate and assayed at two dilutions. Data are presented in mg%. Blood draws were performed at the morning of T&A and 4–6 months following surgery also at the morning hours at the pediatric sleep clinic. IGF-1 levels were measured using an immunoradiometric assay after extraction (DSL, Webster, TX, USA). Interassay coefficient of variation (CV) was <14%; data are presented in nmol/L. The markers were measured at the endocrinology and immunology laboratories at the Soroka University Medical Center.

### 2.5. Postsurgical Sleep Evaluation

All parents filled a validated questionnaire given on the day of surgery and at the postsurgical follow-up visit in our pediatric sleep clinic [24]. In brief, it includes eight items scored by the parent in a 0–4 scale (0 = never; 4 = most of the time). Questions included are being hard to awake, witnessed apnea, breathing difficulties, snoring, sweating, mouth breathing, awakening, and restless sleep.

### 2.6. Data Analysis

Results are presented as means ± SD, unless otherwise stated. All analyses were conducted using statistical software SPPS version 17.0. All numeric data were subjected to statistical analyses with either *t* tests or 2-way analysis of variance procedures for repeated measures, as described by Neuman-Keuls. Post hoc tests were performed as appropriate. A two-tailed *P* < 0.05 was considered statistically significant.

## 3. Results

20 children were enrolled. Four children were lost to followup and were not different than the remaining participants. All 16 children (10 boys and 6 girls) were evaluated before and after T&A. Their mean age at the first encounter was 23 ± 6 months (range 7–33 months), and the mean time of follow-up was 5 ± 2 months (range 3.8–6.2 months).

The demographic, anthropometric, and polysomnographic findings are shown in Table 1.

Most children showed significant improvements in their height (4.81 cm) and weight (1.88 kg) following adenotonsillectomy (average: *P* < 0.001 for both). Thus BMI also increased by 0.85 ± 0.42 kg/m^2^ (*P* = 0.037).

**Figure 1:**
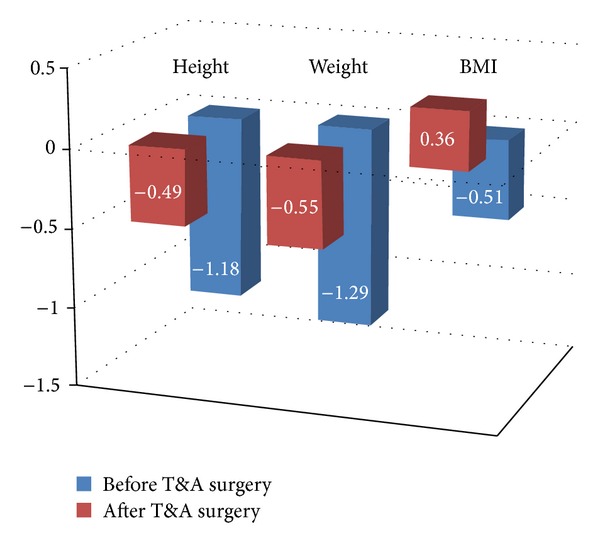
A graphic presentation of the improvement in *Z* score for weight, height, and BMI following T&A surgery. It shows the “catch-up” growth that is demonstrated by the gap of standard deviation to the average values of children of the same age (*Z* score = 0) for weight, height, and BMI.

At diagnosis, mean *Z* scores for height, weight, and BMI were −1.18 ± 0.32, −1.29 ± 0.35, and −0.51 ± 0.15, respectively. After treatment, there were significant improvements in weight *Z* scores (*P* = 0.002) and in BMI *Z* scores (*P* = 0.007) as shown in Figure 1.

IGF-1 and hsCRP circulating levels were improved but did not reach statistical significance. The decrease in systemic inflammation was reflected by decrements of 0.51 ± 0.28 mg% in CRP levels after T&A (*P* = 0.131), while small increases in IGF-1 levels also emerged (2.37 ± 1.41 nmol/L; *P* = 0.104).

**Figure 2:**
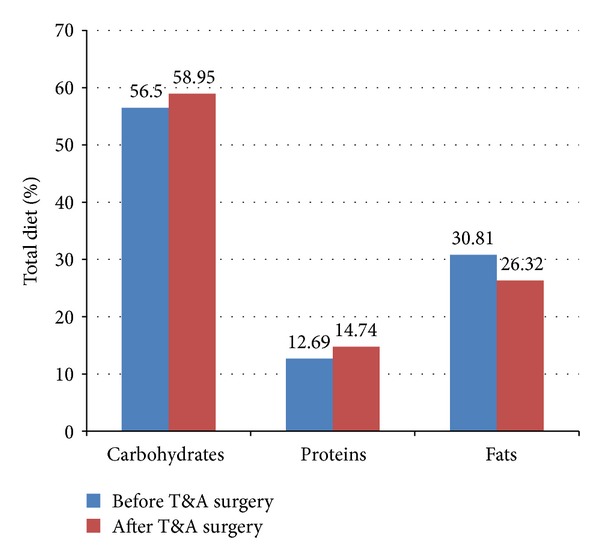
A graphic presentation of the change in dietary composition before and after T&A surgery. A rise in % carbohydrates (NS) and % proteins intake (*P* = 0.045) was noted, accompanied by a decrease in % fat (*P* = 0.016) in diet.

SFFQ were filled by the parents and compared the caloric intake (Table 1). On average, the children added 377.6 ± 292.4 calories to their daily diet after surgery (*P* < 0.001). There were a significant increase in protein intake (2.0 ± 1.4%; *P* = 0.045; Figure 2) and decrease in fat intake (4.49 ± 2.36%; *P* = 0.016). There was also an increase in consumption of carbohydrates of 2% that failed to reach statistical significance (*P* = 0.44).

**Figure 3:**
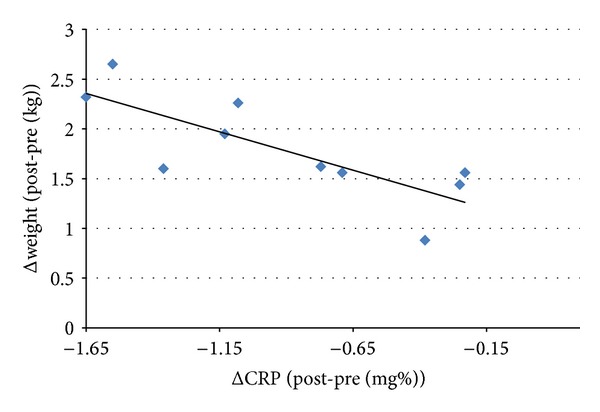
A correlation between the decrease in circulating CRP levels after surgery and the weight the child gained after surgery. The greater the fall in CRP levels the more the weight the boys gained after surgical intervention (*r* = − 0.775, *P* = 0.041).

A multivariate analysis identified a significant negative correlation between systemic inflammation and weight in boys, such that, with more prominent decreases in circulating CRP levels, weight gain following surgery was magnified (*r* = −0.775; *P* = 0.041; Figure 3).

## 4. Discussion

This study found a major improvement in anthropometric parameters accompanied by a decrease in systemic inflammation and an increase in caloric intake and endocrinologic markers following T&A. Furthermore, for the first time it has been proved that even young children with OSA show improvement in somatic growth that is associated in boys with a reduction in systemic inflammation.

This study also identified, for the first time, a significant change in diet composition in children with OSA after surgery. Interestingly, the improvement in somatic growth correlated with an improvement in systemic inflammation but did not correlate with the changes in caloric intake.

We demonstrated that children aged 6–36 months who suffer from sleep disordered breathing are indeed smaller, for height and weight, than their peers before surgery. This gap was minimized following T&A, as shown in the rise in the *Z* scores.

This gap narrowing can be referred to as the “catch-up” growth.

It has been long known that children with OSA improve their somatic growth after surgery, but so far a comprehensive assessment of children 6–36 months was not done. Previous studies that were held in this age group evaluated children for anthropometric parameters before and after T&A and found an improvement of somatic growth [11, 12]. None of them tried to measure circulating levels of endocrine factors or the presence of systemic inflammation and their effect on growth. Furthermore, no dietary assessment has ever been performed in this age group and no one tried to estimate how surgical intervention affects the diet composition.

Several mechanisms have been proposed during the years to try and explain growth retardation in children with OSA. Among them are the presence of systemic inflammation that is well documented in children with OSA [8, 14, 25] and dysphagia from hypertrophied tonsils that serve as a mechanical barrier and obstruct food entry, thus reducing caloric intake [26]. One of the day symptoms of OSA is hyperactive behavior which results in increased motor activity that increases energy expenditure, thus contributing to growth failure [27]. Another possible mechanism is interruption to sleep architecture that interferes with growth hormone secretion [13].

This study tried to find the factor that imposes the biggest influence on “catch-up” growth in these children. Circulating CRP and IGF-1 levels were measured to estimate level of systemic inflammation and growth hormone-insulin-like growth factor 1 (GH-IGF-1) axis, respectively. In order to assess caloric intake, parents completed a Short Food Frequency Questionnaire (SFFQ) for energy assessment for each child on both encounters. This SFFQ is based on a list of items reported by 160 mothers of infants at the age of 6 to 24 months describing the previous day's food items offered or consumed and was validated in previous studies [22, 23].

Other studies that assessed the relationship between nutrition and T&A surgery did show a rise in carbohydrates consumption after T&A, but these studies used 24-hour dietary-recalls and were performed in preschool children [28]. This is the first study that performed dietary assessment in toddlers with OSA before and after T&A using a validated questionnaire. Furthermore, this SFFQ evaluates meal compound during the month prior to each encounter and is less prone to recall bias.

Analyzing the SFFQs showed that all children but one increased their caloric intake after surgical intervention. Moreover, looking at their dietary compound, a rise of 2% in protein and a decline of 4.49% in fat consumption were significantly shown. A nonsignificant rise of 2% percent in carbohydrates was also noticed. Although these changes were found to be significant, their actual effect on growth pattern is not clear, and there is certainly a need to repeat the SFFQ in a larger group in order to draw conclusions.

In parallel to the rise in caloric intake, the children narrowed the weight gap between them and their peers. These two findings can be linked because T&A surgery removes the hypertrophied tissue of tonsils and adenoids, thus removing a mechanical barrier that limits food swallow [19]. This barrier causes dysphagia and obstructs food entry hence causing a lower caloric intake. Removing this barrier enables the child to eat and swallow more easily and increases his caloric intake according to physiologic requirements and gaining weight accordingly [20].

In addition, the change in diet compound not only might indicate hormonal changes that influence appetite but also may represent a rise in anabolic process in the body that is manifested by weight gain. Though being statistically significant, these changes in dietary compound are not marked enough to explain the rise in somatic growth. This should be studied on a larger cohort.

Another possible explanation to the improvement in weight is the decline in systemic inflammation. Previous studies in children with OSA reported high circulating levels of inflammatory factors such as CRP. These inflammatory factors seemed to normalize and improved after surgical intervention. Those studies showed a decline of up to 50% in CRP levels after T&A [14, 25]. We hypothesized that an improvement in systemic inflammation will be followed by an improvement in somatic growth. There was an average decrease of 0.535 mg% in CRP level after surgery; however, the change was not significant probably due to small sample and this should be studied on a larger cohort.

Additional explanation is the improvement in GH-IGF-1 axis. GH mediates growth and is secreted in a pulsatile fashion. Its secretion is increased during sleep, especially slow-wave sleep. IGF-1 mediates the effects of growth hormone in tissues, especially the bone, and promotes its growth. Previous studies reported on shortening of the relative part of slow-wave sleep and improvement in its relative part after T&A [13]. A meta-analysis that tried to determine the role of T&A on GH-IGF-1 axis did find an increase in IGF-1 and IGFBP-3 serum levels, but all the studies were conducted on older children [29]. This study tried to assess whether toddlers will raise their IGF-1 levels after surgery. The children showed an increase in IGF-1 levels after T&A. This may explain the improvement in their weight but it was not significant probably due to a small sample.

In order to find the factor that imposes the biggest influence on “catch-up” growth in OSA children, a multivariate analysis was performed. There was a significant negative correlation between systemic inflammation and weight change in boys. The more prominent the decrease in circulating CRP levels was, the more weight was gained by the child. In contrast, there was no significant correlation between caloric intake and weight. This might support the assumption that the major factor influencing somatic growth in children with OSA is systemic inflammation and not the increase in caloric intake* per se*. The exact role in which systemic inflammation influences growth is not clear. One hypothesis is that local mediators effect bone growth, similar to children with chronic kidney disease [18]. The growth hormone (GH)/insulin-like growth factor 1 (IGF-1) pathway is anabolic to the skeleton and inflammatory cytokines compromise bone growth through a number of different mechanisms, which include interference with the systemic as well as the tissue-level GH/IGF-1 axis. It is known that suppressor of cytokine signaling 2 (SOCS2) expression is increased in inflammatory conditions including CKD and is a recognized inhibitor of GH signaling [18].

Therefore, SOCS2 signaling represents a critical pathway in growth plate chondrocytes through which OSA's activated proinflammatory cytokines alter both GH/IGF-1 signaling and cellular function.

Our report is the first to note the correlation between enhanced somatic growth and decreased systemic inflammation following surgery. It corroborates previous observations regarding other OSA's related morbidities (cardiovascular and neurocognitive) that improved following surgery and correlated with decreased systemic inflammation [8, 30].

Finally, although our findings may be of interest, there is a need to address the limitations of our study.

This report is based on a group of 16 children. Although they were well studied, there may be bias in the outcomes due to the small sample size. Second, we did not assess the resolution of OSA in a repeated PSG. The questionnaire we used shows that the overall symptom improvement was very good (all 8 questions with a scale of 1–5: preop 3.7 ± 1.3; postop 1.3 ± 0.4; *P* = 0.002), but questionnaires can be limited. The sleep disordered breathing scale predicts polysomnographic results to an extent useful for research but it is not always reliable enough for individual patients. However, the sleep disordered breathing scale may predict OSA-related neurobehavioral morbidity and its response to adenotonsillectomy as well as or better than polysomnography [31].

We also need to point out the fact that the children studied represent very accurately the average child in our lab in terms of BMI but not in other countries. Although we also see obese children in our sleep lab, most of the young children are normal or underweight for their age [8]. Therefore, the population recruited in our study may not be representative of current trends in the United States, for example, but still reflects what pediatric sleep specialists encounter in many other countries.

In summary, this study assessed the effect of potential mechanisms on growth in young children treated for OSA. Moreover, it tried to identify the major factor that influences this “catch-up” growth. We found that young children improve their anthropometric measures substantially following surgical removal of their lymphadenoid tissues. We have learned that the increase in caloric intake is accompanied by a change in the dietary composition, that is, an increase in protein and a decrease in fat consumption. The most prominent result is the degree to which T&A changes in systemic inflammation as reported by hsCRP to correlate at least in boys with the degree of catch-up weight gain. This may point to the important role systemic inflammation plays in the growth processes of children with OSA.

## Figures and Tables

**Table 1:** Pre- and postsurgery characteristics.

	Before surgery	After surgery	*P* value
Age (m)	23 ± 6	28 ± 6	<0.001
Time to follow-up (m)		5 ± 2	NA
Weight (kg)	11.11 ± 2.59	13 ± 2.48	<0.001
*Z* score weight (STD)	−1.29 ± 1.84	−0.55 ± 1.51	0.002
Height (cm)	83.28 ± 8.74	88.09 ± 6.34	<0.001
*Z* score height (STD)	−1.18 ± 1.9	−0.49 ± 1.44	0.223
BMI (kg/m^2^)	15.85 ± 1.67	16.7 ± 1.69	0.037
*Z* score BMI (STD)	−0.51 ± 1.24	0.36 ± 1.18	0.007
Circulating CRP (mg%)	0.72 ± 0.9	0.21 ± 0.02	0.131
Circulating IGF-1 (nmol/L)	4.32 ± 2.71	6.69 ± 2.69	0.104
Daily caloric intake (kcal)	837 ± 233	1214 ± 412	<0.001
Apnea-hypopnea index (events/h)	16.8 ± 7.7	NA	
Oxygen minimal (nadir) saturation (%)	78.6 ± 8.4	NA	
Arousals index (events/h)	18.6 ± 11.3	NA	
Total sleep time (min)	429.6 ± 174.3	NA	
